# Miro1 Impairment in a Parkinson’s At-Risk Cohort

**DOI:** 10.3389/fnmol.2021.734273

**Published:** 2021-08-09

**Authors:** David Nguyen, Vinita Bharat, Devon M. Conradson, Pawan Nandakishore, Xinnan Wang

**Affiliations:** ^1^Department of Neurosurgery, Stanford University School of Medicine, Stanford, CA, United States; ^2^Colaberry Inc, Boston, MA, United States

**Keywords:** Parkinson’s disease, IPSC, mitophagy, risk, Miro1

## Abstract

There is a lack of reliable molecular markers for Parkinson’s disease (PD) patients and at-risk individuals. The detection of the pre-symptomatic population of PD will empower more effective clinical intervention to delay or prevent disease onset. We have previously found that the mitochondrial protein Miro1 is resistant to mitochondrial depolarization-induced degradation in fibroblasts from a large number of PD patients and several at-risk individuals. Therefore, Miro1 has the potential to molecularly label PD populations. In order to determine whether Miro1 could serve as a molecular marker for the risk of PD, here we examine the Miro1 response to mitochondrial depolarization by biochemical approaches in induced pluripotent stem cells from a cohort of at-risk individuals. Our results show that the Miro1 phenotype is significantly associated with PD risk. We propose that Miro1 is a promising molecular marker for detecting both PD and at-risk populations. Tracking this Miro1 marker could aid in diagnosis and Miro1-based drug discoveries.

## Introduction

Parkinson’s disease (PD) is the second most common movement disorder, characterized by selective loss of dopaminergic neurons in the substantia nigra. Currently, the diagnosis of PD is based on symptom evaluations with a high probability of misdiagnosis (Dickson, 2018), and there are no reliable biomarkers for PD patients or at-risk individuals, presenting a roadblock to drug development (Bartels, 2016). Early detection in individuals before symptom onset is particularly important, since early intervention will undoubtedly improve treatment efficacy.

**FIGURE 1:**
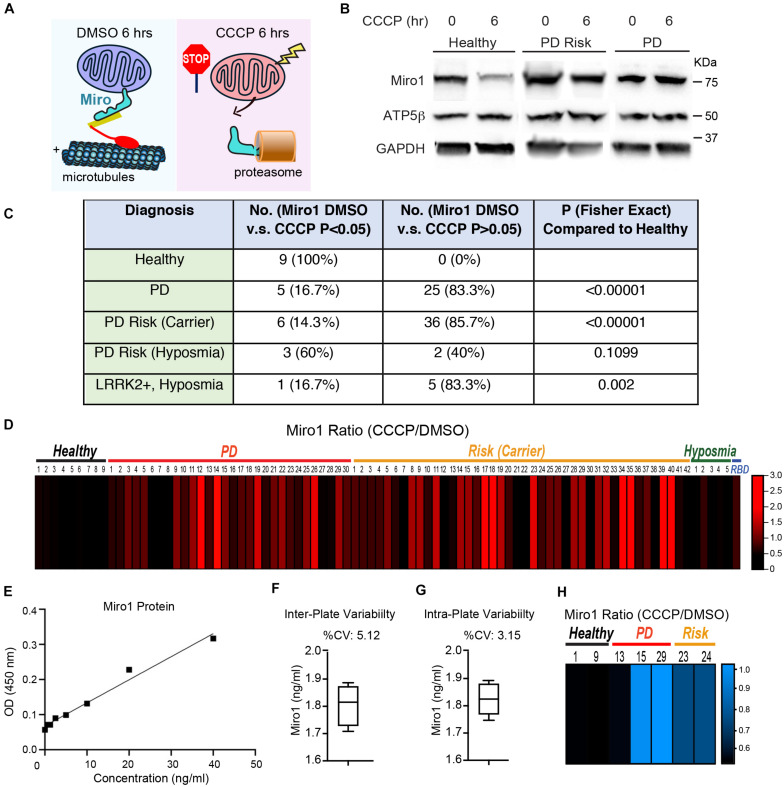
The Miro1 Response to CCCP in iPSCs. **(A)** Schematic representation of our readout. **(B)** Examples of the readout using Healthy-2, PD-29, and Risk-39. Cell lysates were blotted as indicated. **(C)** Summary of the Western blotting results. Normalized Miro1 intensities of “with DMSO” and “with CCCP” within the same subject are compared by Student *T* Test, and the number of subjects with *P* > 0.05 together with subjects that show significant Miro1 upregulation after CCCP, or the number of subjects with *P* < 0.05 for Miro1 reduction after CCCP, is indicated in the column of “No. (Miro1 DMSO vs CCCP *P* > 0.05 or <0.05).” Fisher Exact Test is used to determine the *P* value of a specific group in comparison with “Healthy.” **(D)** The heatmap shows the relative ratio of mean normalized Miro1 intensities (“with CCCP” divided by “with DMSO”) of the same subject measured by Western blotting. Data are from Supplementary Table 1. *n* = 3–55. **(E–G)** Validation of ELISA for Miro1. **(E)** A representative standard curve is shown. LLOD = 0.112 ng/ml. Dynamic range = 0.625–40 ng/ml. **(F)** Inter-plate variability is demonstrated by measuring the same sample (Healthy-1) in 4 different plates. **(G)** Intra-plate variability is shown by measuring the same sample (Healthy-1) 4 times in the same plate. **(H)** The heatmap shows the relative ratio of mean Miro1 values (“with CCCP” divided by “with DMSO”) of the same subject measured by ELISA. Data are from Supplementary Table 1. *n* = 3–4.

We have discovered that a mitochondrial protein, Miro1, is useful for marking a subset of PD patients (Hsieh et al., 2019). Miro1 is attached to the outer mitochondrial membrane and can recruit microtubule motors to mitochondria to mediate their transport (Figure 1A; Guo et al., 2005; Fransson et al., 2006; Wang and Schwarz, 2009). Miro1 is removed from the surface of depolarized mitochondria to arrest their motility and to facilitate their subsequent clearance via mitophagy (Figure 1A; Wang et al., 2011; Hsieh et al., 2016, 2019; Shaltouki et al., 2018). The molecular players that mediate Miro1 removal from damaged mitochondria include Parkinson’s-related proteins—LRRK2, PINK1, and Parkin (Wang et al., 2011; Hsieh et al., 2016). Mutations in *LRRK2*, *PINK1*, or *Parkin* cause familial PD (Kitada et al., 1998; Bonifati, 2002; Valente et al., 2004; Zimprich et al., 2004), and are also associated with the risk of sporadic PD (Cookson, 2010). Therefore, mitophagy may play a key role in Parkinson’s pathogenesis and in additional age-dependent neurodegenerative diseases (Pickrell and Youle, 2015; Fang et al., 2019; Lautrup et al., 2019).

We have previously measured the Miro1 response to mitochondrial depolarization using biochemical assays in skin fibroblasts from a broad spectrum of PD patients, and discovered that 94% of the patients’ fibroblast cell lines fail to remove Miro1 following depolarization (Hsieh et al., 2019). These patients include both familial patients with pathogenic mutations and sporadic patients with no known genetic mutations. Importantly, we have found that the Miro1 defect occurs in five asymptomatic genetic carriers. Those observations suggest that the Miro1 marker may be employed to detect the pre-symptomatic population which will benefit most from early therapeutic intervention. Therefore, it is imperative to determine the frequency of our Miro1 marker in an expansion of non-manifesting genetic carriers, as well as individuals who show prodromal symptoms such as rapid eye movement sleep behavior disorder (RBD) and hyposmia. In the current work, we filled this gap of research by examining our Miro1 marker in induced pluripotent stem cells (iPSCs) from a cohort of genetic carriers and prodromal individuals. These cells represent the entire collection of the first released iPSC repository from Parkinson’s Progression Markers Initiative (PPMI). We discovered a significant association of the Miro1 defect with the risk of PD, yielding important insights into the possibility of detecting the pre-symptomatic phase of this disease.

## Materials and Methods

### Subject Details

No animal models or human subjects were used in this study. The iPSC work was approved by the Stanford Stem Cell Oversight Committee. iPSCs were obtained under an MTA from the National Institute of Neurological Disorders and Stroke (NINDS) human and cell repository or PPMI, which is in a partnership with multiple institutions that deposited iPSCs, approved study protocols, and ensured consent from donors. All iPSC lines in this study are summarized in Supplementary Table 1. PPMI is an international, multi-center, and progressing study designed to identify PD biomarkers by the MJFF^1^. The study design, subject recruitment criteria, site selection, and study assessment have been detailed in Parkinson Progression Marker Initiative (2011).

### Cell Culture and Western Blotting

Induced pluripotent stem cells were cultured in mTeSR Plus Kit (05825, Stemcell Technologies) and maintained in a 37°C, 5% CO_2_ incubator with humidified atmosphere. The media were refreshed every 1–2 days and split every 4–6 days. CCCP (C2759, Sigma-Aldrich) was prepared at 40 mM in DMSO fresh every time and applied at 40 μM in fresh culture medium (1:1000 dilution) for 6 h. Cells were subsequently lysed in NP40 Cell lysis buffer (FNN0021, ThermoFisher Scientific) with protease inhibitor cocktail (539134, Calbiochem). Cell debris was removed by centrifugation at 17,000 *g* for 10 min at 4°C. Cell lysates were mixed 1:1 with 2× Laemmli buffer (4% SDS, 20% Glycerol, 120 mM Tris–HCl, 0.02% bromophenol blue, 700 mM 2-mercaptoethanol) and boiled for 5 min prior to being loaded into an SDS-PAGE. 10% polyacrylamide gels (acrylamide:bis-acrylamide = 29:1) and Tris-Glycine-SDS buffer (24.8 mM Tris, 192 mM glycine, 0.1% SDS) were used for electrophoresis. After electrophoresis, nitrocellulose membranes (1620115, Bio-Rad) were used in semi-dry transfer with Bjerrum Schafer-Nielsen buffer [48 mM Tris, 39 mM glycine, 20% Methanol (v/v), pH 9.2]. Transferred membranes were first blocked overnight in phosphate-buffered saline containing 5% fat-free milk and 0.1% tween-20 at 4°C, and then incubated with the following primary antibodies: mouse anti-Miro1 (WH0055288M1, Sigma-Aldrich) at 1:1,000, mouse anti-ATP5β (AB14730, AbCam) at 1:1,000, or rabbit anti-GAPDH (5174S, Cell Signaling Technology) at 1:1-3,000, at 4°C overnight in blocking buffer. HRP-conjugated goat anti-mouse (115-035-003, Jackson ImmunoResearch Laboratories) or goat anti-rabbit (111-035-144, Jackson ImmunoResearch Laboratories) were used at 1:10-20,000. Pierce ECL Western Blotting Substrate (32109, ThermoScientfic) were used for ECL immunoblotting. Membranes were scanned using a Bio-Rad ChemiDoc XRS system. Experiments were repeated for more than three times. Cell passaging numbers were within the range of 12–17 and did not affect the phenotype.

### Quantification of Western Blotting Data

All experiments were performed in a blinded format. The intensities of protein bands were measured by ImageJ (ver. 1.48V, NIH). The intensity of each band was normalized to that of the loading control GAPDH from the same blot, and expressed as a fraction of that of “Healthy-1 with DMSO treatment” from the same experiment; this control was included in every independent experiment. Values of Mean ± S.E.M of Miro1 were reported in Supplementary Table 1. The ratio of Miro1 was calculated by dividing the mean of Miro1 intensities treated with CCCP by the mean of Miro1 with DMSO of the same subject, and imported into the heat map. Student *T* Test was performed for comparing normalized Miro1 band intensities within the same subject (“with DMSO” vs “with CCCP”), and *P* values were reported in Supplementary Table 1. The number of subjects with a *P* value > 0.05 together with subjects that showed significant Miro1 upregulation after CCCP, or the number of subjects with *P* < 0.05 for Miro1 reduction after CCCP was counted, respectively, and used in Fisher Exact Test in Figure 1C. *n* = 3–55 independent experiments.

**FIGURE 2:**
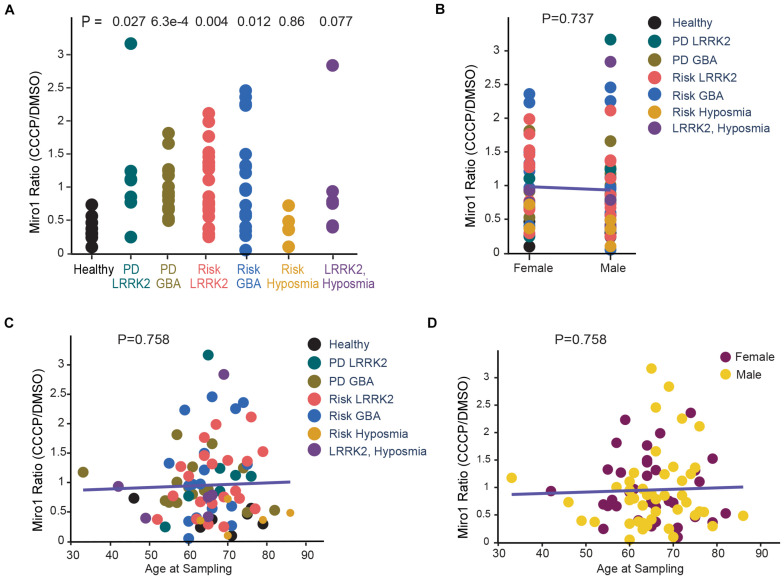
Correlation Analysis of Miro1 Ratio in iPSCs. **(A)** One-Way Anova is used to determine the significant difference among all groups. **(B)** Two-Way Anova is used to determine the interaction between sex and genetic background with Miro1 ratio as the response variable. **(C, D)** Multivariable regression is used to determine the interaction between age and genetic background **(C)**, or age and sex **(D)**, with Miro1 ratio as the response variable. All *P* values are calculated by linear fit.

**FIGURE 3:**
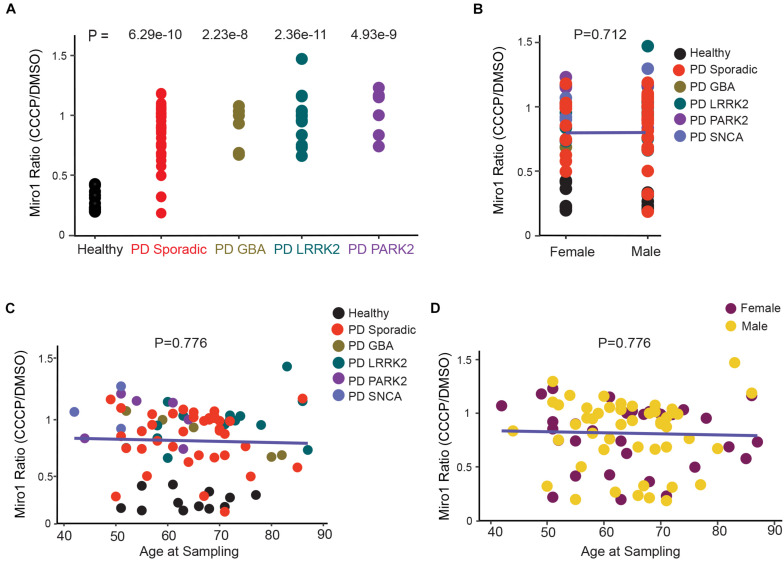
Correlation Analysis of Miro1 Ratio in Fibroblasts. **(A)** One-Way Anova is used. **(B)** Two-Way Anova is used to determine the interaction between sex and genetic background with Miro1 ratio as the response variable. **(C, D)** Multivariable regression is used to determine the interaction between age and genetic background **(C)**, or age and sex **(D)**, with Miro1 ratio as the response variable. All *P* values are calculated by linear fit.

**FIGURE 4:**
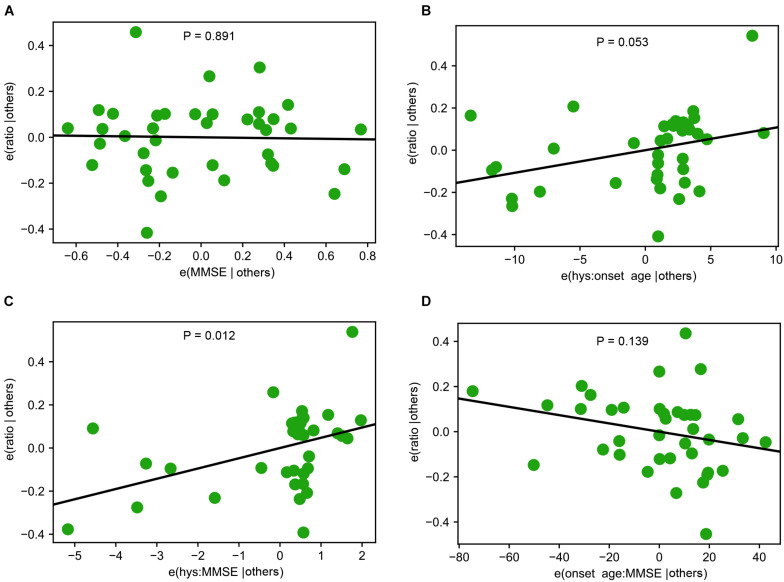
Interactions among Demographic and Clinical Variables. **(A)** A representative partial regression plot shows the influence of a single variable, Mini-Mental Status Examination (MMSE), on Miro1 ratio. **(B–D)** Representative partial regression plots show the interactions of two variables for influencing Miro1 ratio. **(B)** Hoehn and Yahr Scale (hys) and onset age. **(C)** MMSE and hys. **(D)** Onset age and MMSE.

Multivariance regression or Anova was used to determine the interactions among multiple variables for affecting Miro1 ratio and *P* values were calculated by linear fit in Figures 2, 3. During the analysis, Hoehn and Yahr Scale and Mini-Mental Status Examination were detected to show an interaction. Partial regression plots were subsequently generated to help decipher the relationship between an individual variable and the response variable in a multivariable regression problem. Seven partial regression plots, one for each individual variable in the regression problem (Hoehn and Yahr Scale, Mini-Mental Status Examination, onset age), their interaction terms, and the intercept were generated. Out of all the partial regression plots for interaction terms, the plot of Hoehn and Yahr Scale and Mini-Mental Status Examination with linear fit showed significance (*P* = 0.012). This plot and additional representative partial regression plots were shown in Figure 4.

### Enzyme-Linked Immunosorbent Assay

All experiments were performed as blinded tests. 40 μM CCCP in DMSO or the same volume of DMSO alone was applied to iPSCs for 6 h, and then cells were lysed in NP40 Cell lysis buffer (FNN0021, ThermoFisher Scientific) with protease inhibitor cocktail (539134, Calbiochem). Cell debris was removed by centrifugation at 17,000 *g* for 10 min at 4°C. The Rhot1 ELISA kit (EKL54911, Biomatik) was used according to the manufacturer’s instructions. The specificity and stability were validated by Biomatik. The dynamic detection range, sensitivity (lower limit of detection–LLOD), and precision (inter- and intra-assay) were determined by both Biomatik and us (Figures 1E–G), and the results were comparable. Briefly, 50 μl of cell lysate prepared from above, or serial dilutions of the standard (0–40 ng/ml) were added and incubated for 2 h at 37°C. Each well was then incubated with 100 μl of Detection Reagent A for 1 h at 37°C. Next, plates were washed, and each well was incubated with 100 μl of Detection Reagent B for 1 h at 37°C. Plates were washed again, and 90 μl of Substrate Solution was added to each well for 15–25 min at 37°C. The colorimetric reactions were stopped by 50 μl of Stop Solution and absorbance was read at 450 nm by a microplate reader (Infinite 200 Pro, Tecan). An experiment for generating the standard curve was included in each plate and the representative standard plot was shown in the figure. Each data point was from 3 to 4 independent experiments with 2 technical repeats each time. Student *T* Test was performed for comparing Miro1 signals within the same subject (DMSO vs CCCP). A loading control, GAPDH, was detected by Western blotting in each experiment and there was no significant difference in loading between “DMSO” and “CCCP” for each cell line. Raw data and *P* values are reported in Supplementary Table 1.

### Statistics

Throughout the paper, the distribution of data points was expressed as box-whisker or dot-plot, except otherwise stated. Statistical analyses were performed using Prism, Excel, or Python’s statsmodels package. For all experiments, between 3 and 55 independent experiments were performed. The number of experimental replications (n) can be found in Figure Legends and Supplementary Table 1. We did not exclude any data.

## Results

### Miro1 Is Resistant to Degradation in iPSCs From Individuals at Risk for PD

We employed the same methods we previously utilized in fibroblasts that detected Miro1 degradation following mitochondrial depolarization (Hsieh et al., 2019). We cultured iPSCs and applied CCCP, a mitochondrial uncoupler, to depolarize the mitochondrial membrane potential (ΔΨm; Hsieh et al., 2016, 2019). In healthy controls at 6 h following CCCP treatment, Miro1 was significantly degraded as detected by Western blotting (Figures 1A,B); this time point is prior to the completion of mitophagy when multiple mitochondrial markers are degraded (Hsieh et al., 2016, 2019). We applied this method to a total of 87 iPSC lines we obtained from the PPMI and NINDS human and cell repository (Supplementary Table 1). This cohort included 9 wild-type controls (8 healthy subjects and 1 corrected wild-type), 30 PD patients bearing mutations in *SNCA*, *LRRK2*, or *GBA* without the presence of signs for other neurological disorders, 42 asymptomatic genetic carriers (named “Risk”), and 6 individuals exhibiting prodromal symptoms such as hyposmia or RBD but without PD diagnosis (named “Risk-Hyposmia” and “Risk-RBD,” respectively; Supplementary Table 1). 57 individuals have a positive family history. We performed our experiments in a blinded manner. Cell passaging numbers were within the range of 12–17 which had no influence on the phenotype. Notably, we discovered a unifying impairment in degrading Miro1 at 6 h after CCCP treatment in 25 PD (83.3%) and 36 Risk (genetic carriers) lines (85.7%; Figure 1C and Supplementary Table 1). By contrast, Miro1 was efficiently removed following depolarization in every single control subject (0%; Figure 1C and Supplementary Table 1). This phenotype was clearly demonstrated when we imported Miro1 ratio of each individual (Miro1 intensities “with CCCP” divided by “with DMSO”) into a heat map. The majority of the PD and at-risk subjects showed high Miro1 ratios whereas all heathy subjects displayed low Miro1 ratios (Figure 1D). The frequency of the Miro1 phenotype in iPSCs was significantly higher in patients and non-manifesting carriers than healthy subjects (Figure 1C). Interestingly, at-risk individuals with hyposmia alone failed to show a statistical difference from healthy controls, but individuals who were positive for both *LRRK2* mutations and hyposmia had a significantly higher rate of the Miro1 phenotype (Figure 1C). We next validated the results from Western blotting with an alternative method: Enzyme-Linked Immunosorbent Assay (ELISA; Hsieh et al., 2019; Figures 1E–G). We examined seven cell lines used in Figure 1D. For each individual line, the ELISA result of the Miro1 response to mitochondrial depolarization was consistent with that from Western blotting (Figures 1D,H and Supplementary Table 1), demonstrating the robustness of both methods for detecting Miro1 in iPSCs. Similarly, we have previously found that both Western blotting and ELISA are reliable for detecting Miro1 in fibroblasts (Hsieh et al., 2019). Therefore, we have established two methods to measure Miro1 in patients’ cells which could be useful for clinical practice. Miro1 ratio (Miro1 intensities “with CCCP” divided by “with DMSO”) was also significantly correlated with PD and genetic risk (Figure 2A), but not with age (at sampling) or sex (Figures 2B–D). There were no interactions among age, sex, and genetic background for affecting Miro1 ratio (Figures 2B–D). Taken together, these observations show that the failure to remove Miro1 following mitochondrial depolarization is a common cellular defect in this cohort of at-risk individuals.

### Comparison of the Miro1 Defect Between PD Patients and At-Risk Individuals

Having assembled a large dataset of the Miro1 marker in both iPSCs (this paper) and fibroblasts (Hsieh et al., 2019) from PD patients and non-manifesting genetic carriers (Figures 1, 2 and Supplementary Table 1; Hsieh et al., 2019), we performed cross-sectional analyses of the overall outcome. We first compared the frequency of the Miro1 defect in total PD patients between this cohort using iPSCs and the previous cohort using fibroblasts (Hsieh et al., 2019), and found that it was largely consistent (83.3% in iPSCs, 94% in fibroblasts). We next examined the frequency in specific subgroups (sample size ≥ 5; Figures 2A, 3A). The rate of the Miro1 defect was slightly lower in PD patients bearing mutations in *LRRK2* or *GBA* in this cohort using iPSCs than that in the previous cohort using fibroblasts (iPSCs: 83.3 and 93.3%; fibroblasts: 100 and 100%, for *LRRK2* and *GBA*, respectively). We then compared the occurrence of the Miro1 phenotype in iPSCs from PD patients and asymptomatic genetic carriers harboring mutations in the same gene. Notably, non-manifesting genetic carriers bearing mutations in *LRRK2* or *GBA* showed a similar rate of the Miro1 defect as symptomatic patients carrying mutations in the same gene (carriers: 86.4 and 83.3%; PD: 83.3 and 93.3%, for *LRRK2* and *GBA*, respectively).

Lastly, we examined whether there were interactions among genetic background, demographics, and clinical manifestations of PD patients for influencing the Miro1 phenotype using the Western blotting data from fibroblasts we previously published in Hsieh et al. (2019), given the large sample size of this PD cohort (12 healthy subjects and 71 PD patients). Although each individual variable alone including age, sex, Unified PD Rating Scale, Hoehn and Yahr Scale, and Mini-Mental Status Examination did not affect Miro1 ratio, there was a significant interaction between Hoehn and Yahr Scale and Mini-Mental Status Examination (Figures 3B–D, 4), suggesting that Miro1 ratio might respond to PD progression combined with cognitive impairment.

## Discussion

The lack of reliable molecular biomarkers for PD hinders the development of an effective treatment. Although the signature symptoms of PD occur late in life in most patients, the underlying molecular changes and neuronal cell death may have started years prior to symptom onset. Therefore, the identification of a molecular marker that defines the pre-symptomatic population will enable more effective therapeutic intervention. In this study, we have explored the mitochondrial protein Miro1 as a common molecular signature for detecting the pre-disease-onset phase of PD. We have discovered a significant association of the Miro1 defect with PD and its risk using iPSCs, mirroring our previous discovery using fibroblasts (Hsieh et al., 2019). Our findings demonstrate the translational value of Miro1 for marking a subset of Parkinson’s symptomatic and asymptomatic individuals.

The frequency of the Miro1 marker is comparable between non-manifesting genetic carriers and PD patients with mutations in the same gene (*LRRK2* or *GBA*). Given the incomplete penetrance of these mutations (Healy et al., 2008; Marongiu et al., 2008; Nishioka et al., 2009; Anheim et al., 2012), longitudinal studies will be especially valuable to determine the disease conversion rate of at-risk individuals who test positive for the Miro1 defect. Our analyses also detect an interaction between Hoehn and Yahr Scale and Mini-Mental Status Examination for influencing the Miro1 phenotype in PD patients (Figure 4), suggesting that Miro1 could be utilized to monitor PD progression in combination with cognitive impairment. Interestingly, the Miro1 defect occurs in several individuals with hyposmia or RBD. Compared with genetic carriers, the sample sizes for RBD and hyposmia are much smaller, due to the unavailability of sufficient donors. Future work is needed to examine our Miro1 marker in larger cohorts of RBD and hyposmia. It will be similarly beneficial to expand the sample size for genetic carriers and to examine the Miro1 phenotype in additional neurodegenerative diseases. Our work opens new avenues to detecting and treating the disease by using Miro1 as a marker and target, but also raises important questions about the mechanisms underlying the Miro1 impairment in distinct types of PD and the downstream consequences. Our previous work has mechanistically linked depolarization-triggered Miro1 degradation and mitophagy to several PD-causing genes, including *PINK1*, *Parkin*, *LRRK2*, and *SNCA* (Wang et al., 2011; Hsieh et al., 2016, 2019; Shaltouki et al., 2018). Further investigations are warranted to reveal the interplay of Miro1 with additional PD-associated genes such as *GBA*, and the Miro1-dependent pathways in sporadic PD. A systemic characterization of Miro1 protein levels and response to mitochondrial damage with age in heathy and diseased cells will also shed light on Miro1’s role in normal and pathological aging.

## Data Availability Statement

The original contributions presented in the study are included in the article/Supplementary Material; further inquiries can be directed to the corresponding author/s.

## Author Contributions

DN, VB, and DC designed and performed the experiments. DN, VB, and PN analyzed the data, made the figures, and wrote the manuscript. XW conceived and supervised the project, designed the experiments, and wrote the manuscript. All authors contributed to the article and approved the submitted version.

## Conflict of Interest

PN is employed by Colaberry Inc. XW is a co-founder, adviser, and shareholder of AcureX Therapeutics Inc, and a shareholder of Mitokinin Inc. The remaining authors declare that the research was conducted in the absence of any commercial or financial relationships that could be construed as a potential conflict of interest.

## Publisher’s Note

All claims expressed in this article are solely those of the authors and do not necessarily represent those of their affiliated organizations, or those of the publisher, the editors and the reviewers. Any product that may be evaluated in this article, or claim that may be made by its manufacturer, is not guaranteed or endorsed by the publisher.

## Footnotes

1
http://www.ppmi-info.org/study-design/
